# P-507. Implementation of Guideline-Based HIV Prevention at an Academic Primary Care Clinic

**DOI:** 10.1093/ofid/ofae631.706

**Published:** 2025-01-29

**Authors:** Isaac Daudelin, Eileen Kim, Arthi Palani, Ritik Goyal, Heather M Wang, Shobha Swaminathan, Jared Walsh, Diana Finkel

**Affiliations:** Thomas Jefferson University, newark, New Jersey; Rutgers NJMS, Newark, New Jersey; Rutgers NJMS, Newark, New Jersey; Rutgers NJMS, Newark, New Jersey; Rutgers NJMS, Newark, New Jersey; Rutgers New Jersey Medical School, Newark, New Jersey; Rutgers NJMS, Newark, New Jersey; Rutgers NJMS, Newark, New Jersey

## Abstract

**Background:**

Primary care providers often lack familiarity with pre-exposure prophylaxis (PrEP) for HIV and miss screening for high-risk behavior. From 2017-2019 the Academic Internal Medicine Clinic (AIMC) at University Hospital in Newark, NJ averaged 2 PrEP prescriptions/year. Multiple resident lectures to improve screening and treatment saw minimal improvement to 4 PrEP prescriptions/year from 2020 to 2021, all of which were refills. This QA/QI project uses Epic smart tools to speed integration of guideline directed HIV prevention at AIMC.

**Figure:**
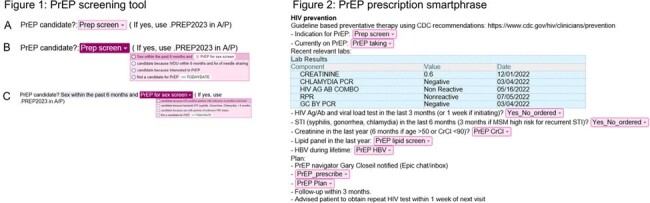

**Methods:**

Intervention: A PrEP screening smartlist was created in Epic and implemented in the common healthcare maintenance (HCM) smartphrase used by residents at AIMC. A positive screen directs the user to another smartphrase which guides the user to start or continue PrEP safely.

Assessment: 2021 CDC HIV PrEP Guidelines were used. Charts from 100 patients randomly selected from 5320 patients visiting AIMC in 2023 were reviewed for documentation of PrEP screening. Epic was queried and charts were reviewed to find all patients prescribed PrEP by AIMC residents in 2023.

**Figure:**
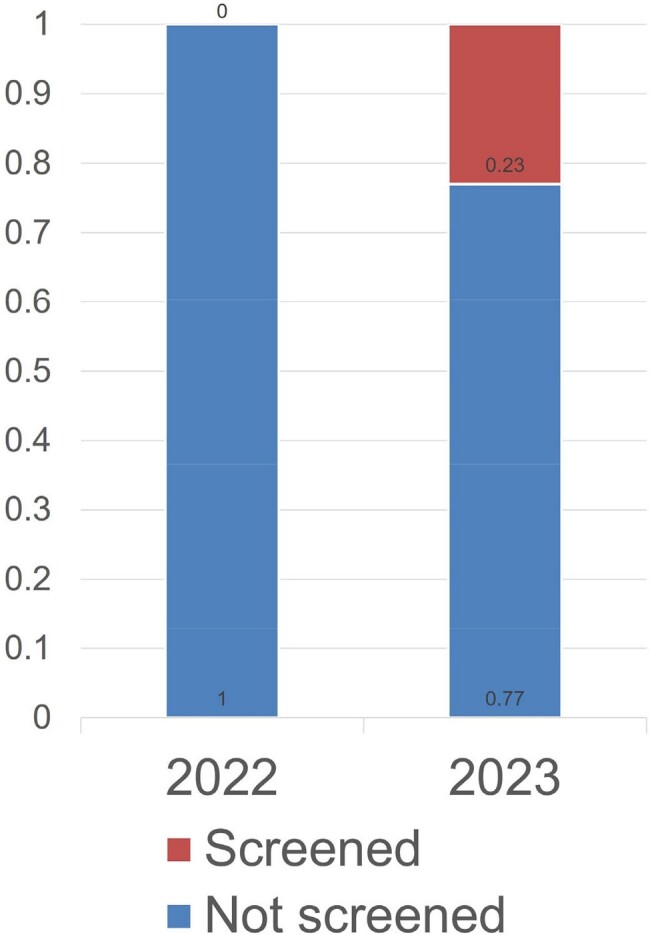

**Results:**

Of patients seen by residents at AIMC in 2023, 23% were screened for PrEP compared to 0% in previous years. 11 unique patients were prescribed PrEP, 5 of which were new prescriptions.

22% of patients seen in 2023 were new to the clinic. Of these, 52% used the common HCM smartphrase, 38% used a personal HCM smartphrase, and 10% used outdated versions of the common HCM smartphrase.

When the common HCM smartphrase was used, 72% of users screened for PrEP, 8% deferred screening to a future visit, and 20% removed the screening tool.

**Figure:**
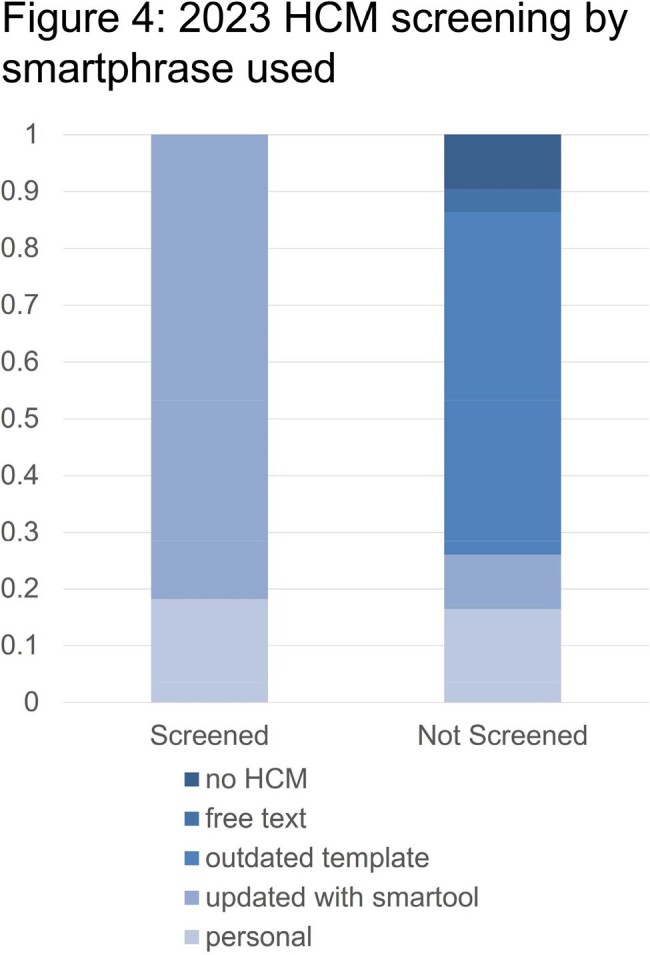

**Conclusion:**

This study shows that an intuitive smart tool can create immediate change in a resident training program. Outdated versions of HCM copied forward from old visits were the largest barrier to this initiative, however this should spontaneously improve each year with 22% of patients in the year new to the clinic. Future directions include: tool use training, email reminders to use the smartphrase or to update personal templates annually to include updated guidelines, resident champions, and/or resident surveys.

**Figure:**
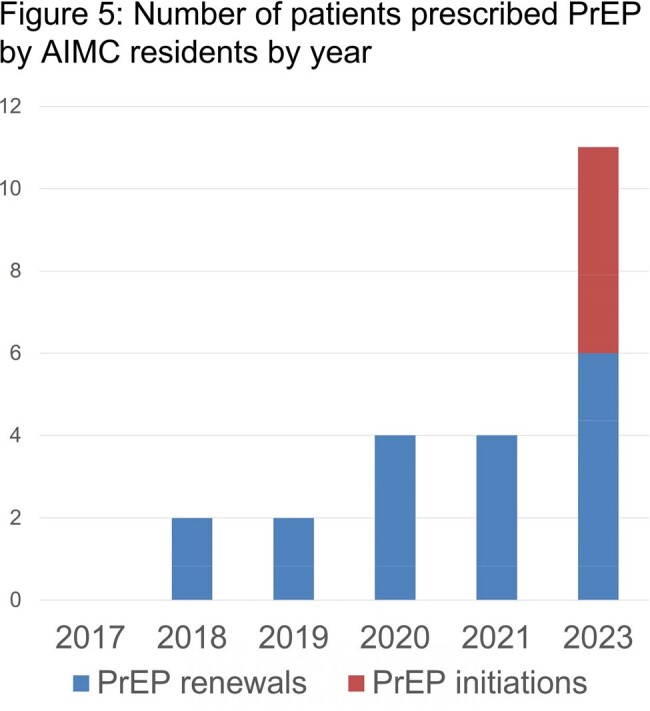

**Disclosures:**

**Shobha Swaminathan, MD**, Viiv Healthcare: Advisor/Consultant

